# The effects of ecstasy on neurotransmitter systems: a review on the findings of molecular imaging studies

**DOI:** 10.1007/s00213-016-4396-5

**Published:** 2016-08-28

**Authors:** Yosta Vegting, Liesbeth Reneman, Jan Booij

**Affiliations:** 1 Department of Nuclear Medicine, Academic Medical Center, University of Amsterdam, 1105 AZ Amsterdam, The Netherlands; 2 Department of Radiology, Academic Medical Center, University of Amsterdam, 1105 AZ Amsterdam, The Netherlands; 3 Brain Imaging Center, Academic Medical Center, University of Amsterdam, 1105 AZ Amsterdam, The Netherlands

**Keywords:** 3,4-Methylenedioxymethamphetamine, MDMA, Ecstasy, Neuroimaging, PET, SPECT, fMRI, Neurotoxicity, Serotonin, Dopamine

## Abstract

**Rationale:**

Ecstasy is a commonly used psychoactive drug with 3,4-methylenedioxymethamphetamine (MDMA) as the main content. Importantly, it has been suggested that use of MDMA may be neurotoxic particularly for serotonergic (5-hydroxytryptamine (5-HT)) neurons. In the past decades, several molecular imaging studies examined directly in vivo the effects of ecstasy/MDMA on neurotransmitter systems.

**Objectives:**

The objective of the present study is to review the effects of ecstasy/MDMA on neurotransmitter systems as assessed by molecular imaging studies in small animals, non-human primates and humans.

**Methods:**

A search in PubMed was performed. Eighty-eight articles were found on which inclusion and exclusion criteria were applied.

**Results:**

Thirty-three studies met the inclusion criteria; all were focused on the 5-HT or dopamine (DA) system. Importantly, 9 out of 11 of the animal studies that examined the effects of MDMA on 5-HT transporter (SERT) availability showed a significant loss of binding potential. In human studies, this was the case for 14 out of 16 studies, particularly in heavy users. In abstinent users, significant recovery of SERT binding was found over time. Most imaging studies in humans that focused on the DA system did not find any significant effect of ecstasy/MDMA use.

**Conclusions:**

Preclinical and clinical molecular imaging studies on the effects of ecstasy/MDMA use/administration on neurotransmitter systems show quite consistent alterations of the 5-HT system. Particularly, in human studies, loss of SERT binding was observed in heavy ecstasy users, which might reflect 5-HT neurotoxicity, although alternative explanations (e.g. down-regulation of the SERT) cannot be excluded.

## Introduction

Ecstasy is a common recreationally used psychoactive drug. The name ecstasy refers to the main effects of the drug, because the Greek word “εκστασις” (ekstasis) means “standing out of yourself”. Euphoric feelings and the ability to socialize can be increased after use of ecstasy/3,4-methylenedioxymethamphetamine (MDMA). Moreover, people can experience entactogenic effects and feel extremely connected with others and some even have mild hallucinations (Reynolds 2013). These effects are caused by MDMA, the main content of ecstasy tablets, through a mechanism of enhanced release of the neurotransmitter serotonin (5-hydroxytryptamine (5-HT)) as well as a relatively small release of another monoaminergic neurotransmitter, namely dopamine (2-(3,4-dihydroxyfenyl)-ethaanamine (DA)) (Lyles and Cadet 2003). Although it is well known from animal studies that MDMA administration induces a massive release of 5-HT and that frequent administrations of MDMA may induce neurotoxic effects on the 5-HT system (Commins et al. 1987; Lyles and Cadet 2003), administration of MDMA may also induce changes on other neurotransmitter systems. Indeed, Battaglia et al. (1988) showed that MDMA has non-negligible affinity for not only 5-HT_1_ and 5-HT_2_ receptors, but also α_1_-adrenergic receptors, α_2_-adrenergic receptors, β-adrenergic receptors, muscarinic M_1_ and M_2_ receptors, histamine H_1_ receptors, DA D_1_ and D_2_ receptors, opioid receptors and benzodiazepine receptor sites.

With the use of molecular neuroimaging techniques like single-photon emission computed tomography (SPECT) and positron emission tomography (PET) imaging, neurotransmitter systems in the living brain can be visualized and specific receptors/transporters quantified, both in laboratory animals, in non-human primates and in humans. Several human molecular imaging studies indicated that the 5-HT transporter (SERT) binding is decreased in different brain regions of frequent MDMA users (Buchert et al. 2007; McCann et al. 2005; Zhou et al. 1998). However, there is discussion whether this alteration in binding may reflect neurotoxicity. Some experimental studies in rodents and primates indicate that administration of MDMA damages the structural and functional integrity of the 5-HT system. In these studies, immunocytochemistry was used and markers of 5-HT axon degeneration were assessed, e.g. concentrations of 5-hydroxyindoleacetic acid (5-HIAA), 5-HT and the SERT (Battaglia et al. 1987; Commins et al. 1987). Immunocytochemistry showed morphologic evidence of neuronal degeneration due to administration of MDMA (Battaglia et al. 1987; Molliver et al. 1990; O’Hearn et al. 1988; Ricaurte and McCann 1992). In contrast, alternative explanations for the loss of SERT after MDMA administration were put forward as well. It was suggested that the administration of MDMA may cause a state of metabolic exhaustion through a mechanism of modifications in gene expression and protein function (Baumann et al. 2007). This hypothesis is supported by studies that measured glial activation and silver staining, also indicators of neurotoxicity. In these studies, no correlation was found between 5-HT depletion induced by MDMA administration and markers of neurotoxicity in mice treated with 10–20 mg/kg MDMA (Pubill et al. 2003; Wang et al. 2004).

The United Nations Office on Drugs and Crime has estimated that there were 18.8 million ecstasy users worldwide in 2013. From 2009 to 2013, a decrease was found in the prevalence of ecstasy use in the past year in subjects of 15 to 64 years (United Nations Office on Drugs and Crime 2015). However, the average amount of MDMA in an ecstasy tablet in the Netherlands has increased over the years (Van Laar et al. 2015; Vogels et al. 2009). Therefore, the amount of MDMA administered within a short time frame may have risen.

Although in the last 10 years, the average dosage of MDMA in ecstasy tablets has increased, potential long-term effects of MDMA/ecstasy use remain unclear, most likely because the conducted studies differ in their methodology and findings are thus difficult to compare. It has been suggested that ecstasy use might be a threat for public health (Cowan 2007); however, at the same time, an increased interest in the use of MDMA in a therapeutic setting is being reported, for example, to enhance the effectiveness of psychotherapy in resistant, chronic posttraumatic stress disorder (PTSD) (Oehen et al. 2013). Also, a recently published review of Mueller et al. (2015) did not find convincing evidence from neuroimaging studies that *moderate* use of MDMA is neurotoxic in humans.

To draw conclusions whether MDMA may induce changes in neurotransmitter systems, we offer a review of the results of imaging studies on the effects of ecstasy/MDMA on neurotransmitter systems in small laboratory animals, non-human primates and humans.

## Methodology

### Search and information source

With the search terms stated below (Table 1), a search in the online database PubMed was carried out updated until 14 November 2015. The Patient–Intervention–Comparison–Outcomes (PICO) system (Richardson et al. 1995) was used to construct the search. To increase the sensitivity of the search, finally, only search terms for the intervention with MDMA and search terms for the different imaging techniques were included.

**Table 1:** Search terms in PubMed

((“N-Methyl-3,4-methylenedioxyamphetamine”[Mesh] OR MDMA[tiab] OR Ecstasy[tiab] OR Ecstacy[tiab] OR methylenedioxyamphetamine[tiab] OR N 3,4 Methylenedioxyamphetamine[tiab]) OR Ecstacy*)) AND (“Tomography, Emission-Computed, Single-Photon”[Mesh] OR SPECT[tiab] OR PET[tiab] OR PET scan* OR SPECT scan* OR Single-Photon Emission-Computed Tomograph* OR “Positron-Emission Tomography”[Mesh] OR Positron-Emission Tomograph* OR phMRI[tiab] OR pharmacological MRI[tiab])	

### Selection of studies

**Fig. 1:**
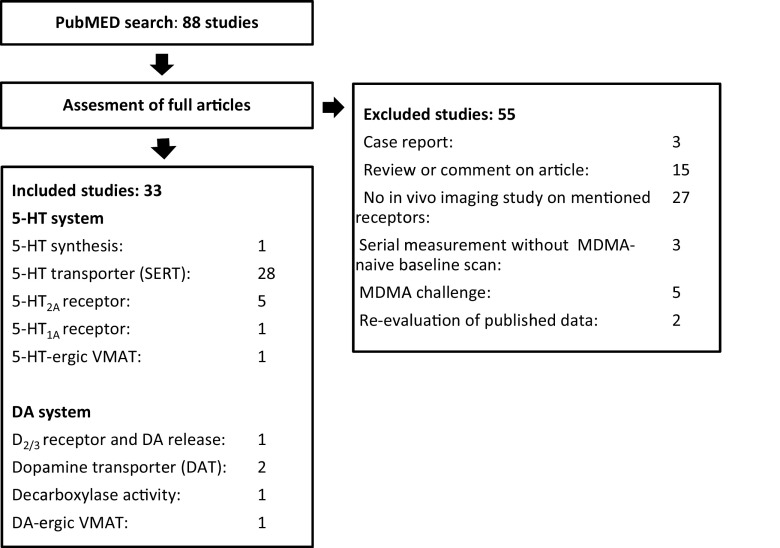
Flowchart of the inclusion and exclusion of studies

Full-text articles were obtained on which inclusion and exclusion criteria were applied. Criteria for selecting the articles were as follows. Publications were included if (1) in vivo imaging findings on neurotransmitter systems were reported and (2) the data was obtained in a control group with an MDMA-naive condition or in a serial measurement in which the baseline measurement (T1) was in a MDMA-naive state. Publications were excluded if (1) the study design was a case report study or a review, (2) MDMA was given as a single challenge, or (3) the study was a re-evaluation of previously published data. Figure 1 shows the flowchart of the inclusion and exclusion of the studies.

### Data extraction

Data was extracted about the (1) receptor/transporter studied, (2) number of participated subjects and controls with key features, (3) radiotracer used, (4) amount of ecstasy use/administration, (5) minimal time of MDMA/ecstasy abstinence and (6) results of the particular study. We extracted and reported *P* values and preferably *P* values that were corrected for multiple comparisons. For the papers that reported means and standard deviations, we calculated the percentage of alteration of tracer binding. We defined an increase or reduction as follows:$\mathrm{Alteration}=100*\frac{\left(\mathrm{imaging}\ \mathrm{outcome}\ \mathrm{measure}\ \mathrm{in}\ \mathrm{MDMA}\ \mathrm{users}-\mathrm{outcome}\ \mathrm{measure}\ \mathrm{in}\ \mathrm{controls}\right)}{\mathrm{outcome}\ \mathrm{measure}\ \mathrm{in}\ \mathrm{controls}}$ and expressed it as a percentage. To estimate the size of the differences found (between the MDMA group and the control condition), we calculated effect sizes (ES), using the Cohen’s *d*. We subtracted the mean of the control group from the mean of the MDMA group, which was divided by the pooled standard deviation as follows: $d=\frac{M\ \mathrm{MDMA}-M\ \mathrm{control}}{SD\ \mathrm{pooled}}$.

## Results

### Inclusion of studies

Eighty-eight studies were found after the initial search in PubMed (Fig. 1). Thirty-three studies were included after applying inclusion and exclusion criteria as mentioned before. The included studies examined the effects of ecstasy on 5-HT synthesis, the SERT, 5-HT_2A_ receptor, 5-HT_1A_ receptor, 5-HT-ergic vesicular monoamine transporter (VMAT; i.e. VMAT expression in 5-HT-rich brain areas), DA D_2/3_ receptor and DA release, the DA transporter (DAT), decarboxylase activity and DA-ergic VMAT (i.e. VMAT expression in DA-rich brain areas).

### Serotonin system

#### 5-Hydroxytryptamine synthesis

In our search, only one human study on 5-HT synthesis was found and included (Table 2). A whole-brain SPM analysis showed decreased 5-HT synthesis in a large brain area, from the prefrontal and orbitofrontal cortex all the way up to the posterior parietal cortex in MDMA polydrug users compared to polydrug using controls (data not in Table 2). Also, increased uptake was observed in the brainstem, in the region of the periaqueductal grey matter, as well as in parts of the left lateral prefrontal cortex and temporal cortex. The volumes of interest  (VOI) analyses, in which gender effects were assessed, showed that 5-HT synthesis levels were significantly increased in the raphe nuclei (raphe, *P* = 0.01, effect size (ES) = 1.43) and tend to be increased in the brainstem in female MDMA polydrug users compared to female controls (Table 2). Furthermore, a significant decreased tracer uptake was found in the lateral orbitofrontal brain area in female MDMA polydrug users as compared to female controls. Male MDMA polydrug users showed lower uptake in the pre-central gyrus compared to male controls (pre-central gyrus, *P* = 0.029, ES = −1.14).

**Table 2:** 5-HT synthesis human studies

Technique used	Author	Nr pts/controls	Inclusion and exclusion criteria	MDMA check	Outcome		Effect size
[^11^C]AMT PET	Booij et al. (2014)	*MDMA*, 17 *PD-Controls*, 18	*MDMA*: ecstasy >25 times	≥3-week abstinent, urine screening	*MDMA vs PD-Controls*:^a^		
			Use of other drugs allowed		*Men:*		
			*PD-Controls*: ecstasy <5 times		Raphe: increase	3 %	0.10
			Use of other drugs allowed		Brainstem: increase	6 %	0.86
					Pre-cuneus: decrease	−1 %	−0.18
			Cannabis average <1 time/month		Pre-central gyrus: decrease (*P* = 0.029)	−6 %	−1.14
					Lateral orbitofrontal gyrus: increase	6 %	0.51
					*Women:*		
					Raphe: increase (*P* = 0.01)	30 %	1.43
					Brainstem: increase	9 %	0.58
					Pre-cuneus: decrease	−1 %	−0.07
					Pre-central gyrus: increase	0 %	0.03
					Lateral orbitofrontal: decrease (*P* = 0.03)	−11 %	−1.14

This table shows the results of human studies on 5-HT synthesis (VOI analyses; data taken from Table 5 in Booij et al. 2014, since these data could be used to calculate effect sizes). Only significant *P* values (not corrected for multiple comparisons) are presented. “MDMA” means MDMA users. “PD-Controls” are polydrug users (excluding MDMA use)

^a^Results are shown of a selection of brain regions

#### Serotonin transporter

Twenty-seven studies were included that studied SERT binding in vivo (Tables 3 and 4). Eleven studies were performed in animals and 16 studies in humans. Importantly, 14 out of 16 of the human studies showed a significant loss of SERT binding, while in animal studies, this was found in 9 out of 11 studies. All over, the ES were larger (ranging from −0.38 to −20.03) in animal studies than in human studies (ranging from −0.05 to −2.17).

**Table 3:** SERT animal studies

Technique used	Author	Nr pts/controls	Details animals	Dosage MDMA	Outcome		Effect size
[¹²³I]β-CIT SPECT	Klomp et al. (2012)	*Adolescent treated rats* MDMA 5 Controls 5 *Adult treated rats* MDMA 8 Controls 8	Wistar rats	*MDMA* 10 mg/kg, 8 doses	*Adolescent-treated MDMA vs controls*:		
					Cortical regions (*P* < 0.01)*		
					Prefrontal cortex: decrease (*P* < 0.01)*	−35 %	−3.11
					Temporal cortex: decrease (*P* < 0.01)*	−27 %	−2.82
					Occipital cortex: decrease (*P* < 0.01)*	−38 %	−4.49
					Subcortical regions (*P* < 0.01)*		
					Amygdala: decrease (*P* < 0.01)*	−38 %	−2.75
					Hippocampus: decreases (*P* < 0.01)*	−38 %	−3.97
					Hypothalamus: decrease	−20 %	−1.32
					Midbrain (sup. coll.): decrease (*P* < 0.01)*	−69 %	−5.00
					Striatum: increase	20 %	0.69
					*Adult-treated MDMA vs controls*:		
					Cortical regions (*P* < 0.01)*		
					Prefrontal cortex: decrease (*P* < 0.01)*	−49 %	−5.47
					Temporal cortex: decrease (*P* < 0.01)*	−37 %	−3.07
					Occipital cortex: decrease (*P* < 0.01)*	−42 %	−2.52
					Subcortical regions (*P* < 0.01)*		
					Amygdala: decrease (*P* < 0.01)*	−45 %	−3.75
					Hippocampus: decrease (*P* < 0.01)*	−41 %	−3.90
					Hypothalamus: decrease (*P* < 0.01)*	−35 %	−2.43
					Midbrain: decrease (sup. coll.) (*P* < 0.01)*	−75 %	−5.62
					Striatum: increase	36 %	1.01
[¹²³I]β-CIT SPECT	de Win et al. (2004a)	*Baseline + MDMA* 4	Male Wistar rats	*MDMA*: day 1–2, 20 mg/kg, 1 dose; day 3, 10 mg, 1 dose; day 4, 10 mg/kg, 2 doses	*MDMA vs baseline*:		
					Striatum: decrease	−5 %	−0.38
					Thalamus: decrease (*P* = 0.044)	−21 %	−1.99
[¹²³I]β-CIT SPECT	Reneman et al. (2002a)	*Baseline + MDMA* 1	Rhesus monkey	*MDMA* 5 mg/kg, 8 doses	*MDMA 10 days vs Baseline*:		
					Hypothalamic/midbrain: decrease	−39 %	^d^
					Striatum: decrease	−13 %	
					*MDMA 31 days vs Baseline*:		
					Hypothalamic/midbrain: decrease	−34 %	
					Striatum: decrease	−26 %	
[^11^C]DASB PET	Beaudoin-Gobert et al. (2015)	*Baseline + MDMA* 7	Macaque monkeys	*MDMA* 5 mg/kg, 8 doses MPTP injections (DA lesion) in history 0.3–0.5 mg/kg	*MDMA vs baseline*:^a^		
					Thalamus: decrease (*P* < 0.001)*	−47 %	−5.26
					Hippocampus: decrease (*P* < 0.01)*	−47 %	−4.21
					Brainstem: decrease (*P* < 0.05)*	−40 %	−4.12
					Occipital cortex: decrease (*P* < 0.05)*	−63 %	−4.12
					Prefrontal cortex: decrease	−63 %	−8.27
[^11^C]DASB PET	Gould et al. (2011)	*SA-MDMA* 4	Male Rhesus monkeys	10–12 months only the exclusive drug *SA-MDMA monkeys*: Lifetime intake MDMA 97–141 mg/kg Lifetime intake Cocaine: <120 mg/kg	*SA-MDMA vs DN-Controls*:		
		*SA-Cocaine* 4					
		*DN-Controls* 4			Thalamus: decrease	−10 %	−1.59
					Amygdala: increase	7 %	2.19
					Hippocampus: increase	7 %	1.58
					Prefrontal cortex: decrease	−10 %	−3.15
					Midcingulate cortex: decrease (*P* < 0.005)*	−9 %	−7.59
					Temporal cortex: decrease	−5 %	−1.17
					Parietal cortex: decrease (*P* < 0.05)*	−11 %	−3.75
					Occipital cortex: decrease	−17 %	−5.82
[^11^C]DASB PET	Banks et al. (2008)	SA-*MDMA* 4	Rhesus monkeys	*SA-MDMA monkeys*: Lifetime intake MDMA 97–141 mg/kg Mean cocaine intake 67 mg/kg	*MDMA vs DN-Controls*:		
		*SA-Cocaine* 4					
		*DN-Controls* 4			Caudate nucleus: decrease	−9 %	−1.58
					Putamen: decrease	−4 %	−0.72
					Anterior cingulate cortex: decrease	−11 %	−3.16
[^11^C]DASB PET	Cumming et al. (2007)	*Baseline + MDMA* 6	12 Göttingen minipigs	*MDMA*: Mean 42 mg/kg	*MDMA vs baseline*:		
					Frontal cortex: decrease (*P* < 0.01)	−61 %	^d^
					Temporal cortex: decrease (*P* < 0.01)	−52 %	
					Occipital cortex: decrease (*P* < 0.01)	−45 %	
					Caudate/putamen: decrease (*P* < 0.01)	−58 %	
					Ventral forebrain: decrease (*P* < 0.01)	−50 %	
					Thalamus: decrease (*P* < 0.01)	−32 %	
					Medial hypothalamus: decrease (*P* < 0.01)	−33 %	
					Mesencephalon: decrease (*P* < 0.01)	−30 %	
					Pons: decrease (*P* < 0.01)	−32 %	
[^11^C]DASB PET	Szabo et al. (2002)	*Baseline + MDMA* 1	Baboon	*MDMA* 5 mg/kg, 4 doses, 17 and 7 months before initiation PET study	*MDMA vs controls*:		
					Pons: decrease	−43 %	−2.44
					Midbrain: decrease	−56 %	−3.81
					Hypothalamus: decrease (*P* < 0.05)	−61 %	−5.48
					Thalamus: decrease (*P* < 0.05) *	−65 %	−9.08
					Putamen: decrease (*P* < 0.05)	−60 %	−5.50
					Caudate: decrease (*P* < 0.05)	−58 %	−7.95
					Frontal cortex: decrease	−38 %	−3.98
					Parietal cortex: decrease (*P* < 0.05)	−36 %	−4.47
					Temporal cortex: decrease	−38 %	−3.98
					Cingulate cortex: decrease (*P* < 0.05)	−40 %	−4.54
					Occipital cortex: decrease (*P* < 0.05)	−33 %	−4.47
					Cerebellum: decrease (*P* < 0.05)	−32 %	−5.09
[^11^C]McN5652 PET					*MDMA vs controls*:		
					Pons: decrease (*P* < 0.05)*	−41 %	−9.20
					Midbrain: decrease (*P* < 0.05)	−44 %	−6.80
					Hypothalamus: decrease (*P* < 0.05)*	−54 %	−9.95
					Thalamus: decrease (*P* < 0.05)*	−57 %	−20.03
					Putamen: decrease (*P* < 0.05)*	−53 %	−11.88
					Caudate: decrease (*P* < 0.05)*	−46 %	−8.67
					Frontal cortex: decrease (*P* < 0.05)*	−36 %	−9.91
					Parietal cortex: decrease (*P* < 0.05)*	−40 %	−12.81
					Temporal cortex: decrease (*P* < 0.05)*	−35 %	−9.43
					Cingulate cortex: decrease (*P* < 0.05)*	−38 %	−13.82
					Occipital cortex: decrease (*P* < 0.05)*	−41 %	−18.66
					Cerebellum: decrease (*P* < 0.05)*	−36 %	−14.14
[^11^C]McN5652 PET	Scheffel et al. (1998)^b^	*Baseline + MDMA* 1	Baboon	*MDMA* 5 mg/kg, 8 doses	*MDMA after 40 days vs baseline*:		
					Pons: sign. decrease*	−53.9 %	^d^
					Hypothalamus: sign. decrease*	−51.3 %	
					Caudate: sign. decrease*	−66.5 %	
					Putamen: sign. decrease*	−74.8 %	
					Frontal: sign. decrease*	−94.5 %	
					Parietal cortex: sign. decrease*	−72.2 %	
					Occipital cortex: sign. decrease*	−88.6 %	
					*MDMA after 13 months vs control*:		
					Pons: increase	85 %	
					Midbrain: increase	101 %	
					Hypothalamus: increase	123 %	
					Cortical areas: remain decreased		
					Frontal cortex: decrease	−62 %	
					Parietal cortex: decrease	−78 %	
					Occipital cortex: decrease	−73 %	
4-[^18^F]ADAM PET	Chen et al. (2012)	*MDMA* 1 *DN-Controls* 6	Monkey	*MDMA* 5 mg/kg, 8 doses	*MDMA after 12 months vs controls*:		
					Midbrain: decrease (*P* < 0.05)	−39 %	−7.97
					Thalamus: decrease (*P* < 0.05)	−32 %	−4.56
					Striatum: decrease (*P* < 0.05)	−30 %	−4.68
					Frontal cortex: decrease (*P* < 0.05)	−41 %	−1.90
4-[^18^F]ADAM PET	Li et al. (2010)	*SAL/MDMA* 6	Male Sprague–Dawley rats	*MDMA* 10 mg/kg, 8 doses	*SAL/MDMA vs controls*: *day 31* ^**c**^		
		*FLU/MDMA* 6			Midbrain: decrease (*P* < 0.01)*	−71 %	^d^
		*DN-Controls* 6			Hypothalamus: decrease (*P* < 0.01)*	−60 %	
					Thalamus: decrease (*P* < 0.01)*	−60 %	
					Caudate putamen: decrease (*P* < 0.01)*	−50 %	
					Hippocampus: decrease (*P* < 0.01)*	−55 %	
					Frontal cortex: decrease (*P* < 0.01)*	−47 %	

This table reports about animal studies on SERT. Only significant *P* values are shown. *P* values corrected for multiple comparisons are marked with the sign *. “Adolescent treated rats” are rats that were treated with MDMA in adolescence (PND27). “Adult treated rats” are rats that were treated with MDMA in adulthood (PND63). “Baseline + MDMA” means that a baseline scan was taken, followed by that MDMA was given and a second scan was taken. “SA-MDMA” is animals that self-administered MDMA. “SA-Cocaine” is animals that self-administered cocaine. “DN-Controls” are drug-naive controls. “MDMA” means that the animals are treated with MDMA. “SAL/MDMA” means that the rats are treated with saline and MDMA. “FLU/MDMA” means that the rats are treated with fluoxetine and MDMA

^a^Results are shown of a selection of brain regions

^b^Additional longitudinal data not shown in the table

^c^Estimated values, data was shown in a graphic

^d^Not all results were shown in the publication; therefore, the effect sizes could not be calculated

**Table 4:** SERT human studies

Technique used	Author	Nr pts/controls	Inclusion and exclusion criteria	MDMA check	Outcome		Effect size
[¹¹C]DASB PET	Frokjaer et al. (2014)	*MDMA* 18 *DN-Controls* 32	*DN-Controls*: drug-naive Cannabis <15 exposures	≥11-day abstinent, urine screening, hair analysis	*MDMA vs DN-Controls*:		
					Prefrontal cortex: decrease (*P* ≤ 0.0001)	−32 %	^j^
					Midbrain: decrease	−2 %	
[¹¹C]DASB PET	Urban et al. (2012)	*MDMA* 13 *DN-Controls* 13	*MDMA*: ecstasy >15 times Occasional recreational use of other drugs allowed *DN-Controls*: drug-naive	≥2-week abstinent, urine screening, hair analysis	*MDMA vs DN-Controls*:		
					Dorsolateral prefrontal cortex: decrease	−83 %	−0.65
					Medial prefrontal cortex: decrease (*P* = 0.04)	−46 %	−0.90
					Orbitofrontal cortex: decrease	−100 %	−0.40
					Temporal cortex: decrease (*P* = 0.04)	−53 %	−0.89
					Medial temporal lobe: decrease	−16 %	−0.47
					Parietal cortex: decrease	−100 %	−0.66
					Occipital cortex: decrease (*P* = 0.01)	−100 %	−1.14
					Anterior cingulate: decrease	−25 %	−0.57
					Insula: decrease	−13 %	−0.39
					Amygdala: decrease	−7 %	−0.26
					Entorhinal cortex: decrease	−32 %	−0.58
					Hippocampus: decrease	−3 %	−0.08
					Uncus: decrease	−16 %	−0.45
					Caudate: no change	0 %	0.00
					Putamen: increase	2 %	0.08
					Thalamus: decrease	−5 %	−0.27
					Midbrain: increase	1 %	0.04
[¹¹C]DASB PET	Erritzoe et al. (2011)	*MDMA-preferring users (MPU)* 14 *Hallucinogen-preferring users (HPU)* 10 *DN-Controls* 21	*MPU or HPU*: ecstasy or hallucinogen >12 times *MPU*: MDMA/hallucinogen number of lifetime exposures ratio >1 *HPU*: MDMA/hallucinogen number of lifetime exposures ratio <1 *DN-Controls*: drug-naive Cannabis <15 exposures	≥1-week abstinent, urine screening, hair analysis	*MPU vs DN-Control*:		
					Pallidostriatum: decrease (*P* = 0.001)*	−19 %	^j^
					Amygdala: decrease (*P* ≤ 0.001)*	−32 %	
					Neocortex: decrease (*P* ≤ 0.001)*	−56 %	
					Orbitofrontal cortex: decrease	−40 %	
					Medial inferior frontal cortex: decrease	−53 %	
					Superior frontal cortex: decrease	−61 %	
					Superior temporal cortex: decrease	−48 %	
					Medial inferior temporal cortex: decrease	−51 %	
					Sensory motor cortex: decrease	−66 %	
					Parietal cortex: decrease	−47 %	
					Occipital cortex: decrease	−73 %	
					Thalamus: decrease		
[¹¹C]DASB PET	Kish et al. (2010)	*MDMA* 49 *DN-Controls* 50	*MDMA* 4 years chronic ecstasy use (1–2 tablets bimonthly) Use of other drugs *DN-Controls*: no drugs except cannabis	11–194 days of reported abstinence, urine screening, hair analysis	*MDMA vs DN-Controls*:		
					Striatum: no change		^j^
					Thalamus: no change		
					Globus pallidus: no change		
					Hippocampus: decrease (*P* ≤ 0.001)*	−31 %	
					Midbrain: no change		
					Cerebral cortices: decrease (*P* ≤ 0.01)*		
					- Occipital: decrease	−39 %	
					- Frontal: decrease	−17 %	
					- Parietal: decrease	−19 %	
					- Temporal: decrease	−34 %	
					- Insular cortex: decrease	−27 %	
					- Cingulate: decrease	−30 %	
[¹¹C]DASB PET	Selvaraj et al. (2009)	*Ex-MDMA* 12 *PD-Controls* 9 *DN-Controls* 19	*Ex-MDMA*: ecstasy >25 times, >1-year abstinence Use of other drugs *PD-Controls*: ecstasy-naive polydrug users *DN-Controls*: drug-naive	≥3-day abstinent for recreational drugs, urine screening, hair analysis	No significant correlations observed between any variables of MDMA use and [^11^C]DASB binding.		^j^
[¹¹C]DASB PET	McCann et al. (2008)	*MDMA* 16 *PD-Controls* 16	*MDMA*: ecstasy >25 times	≥2-week abstinent from all psychotropic drugs and MDMA, urine screening	*MDMA vs PD-Controls*:		
					Midbrain: decrease	−15 %	−0.34
					Amygdala: decrease	−20 %	−0.68
					Hippocampus: decrease (*P* ≤ 0.01)*	−27 %	−1.08
					Thalamus: decrease	−18 %	−0.53
					Caudate: decrease	−13 %	−0.36
					Putamen: decrease	−6 %	−0.15
					DLPF cortex: decrease (*P* ≤ 0.01)*	−38 %	−0.99
					Occipital cortex: decrease (*P* ≤ 0.0001)*	−59 %	−1.85
					Orbitofrontal cortex: decrease (*P* ≤ 0.05)*	−23 %	−0.70
					Parietal cortex: decrease (*P* ≤ 0.001)*	−41 %	−1.44
					Temporal cortex: decrease (*P* ≤ 0.001)*	−35 %	−1.40
					Ant. cingulate cortex: decrease (*P* ≤ 0.01)*	−25 %	−1.09
					Post. cingulate cortex: decrease (*P* ≤ 0.0001)*	−38 %	−1.84
					Dorsal pons: increase	1 %	0.02
					Ventral pons: decrease	−3 %	−0.05
[¹¹C]DASB PET	McCann et al. (2005)	*MDMA* 23 *PD-Controls* 19	*MDMA*: ecstasy >25 times on separate occasions *PD-Controls*: ecstasy-naive polydrug users Both groups used other recreational drugs	≥2-week abstinent, urine screening	*MDMA vs PD-Controls*:		
					Midbrain: decrease	−21 %	−0.49
					Amygdala: decrease (*P* ≤ 0.05)	−26 %	−0.65
					Hippocampus: decrease (*P* ≤ 0.01)	−40 %	−1.27
					Thalamus: decrease (*P* ≤ 0.05)	−23 %	−0.63
					Caudate: decrease	−14 %	−0.42
					Putamen: decrease	−13 %	−0.36
					DLPF cortex: decrease (*P* < 0.0001)	−55 %	−1.66
					Occipital cortex: decrease (*P* < 0.0001)	−68 %	−2.17
					Orbitofrontal cortex: decrease (*P* < 0.001)	−42 %	−1.29
					Parietal cortex: decrease (*P* < 0.0001)	−51 %	−1.86
					Temporal cortex: decrease (*P* < 0.0001)	−48 %	−2.09
					Ant. cingulate cortex: decrease (*P* < 0.001)	−34 %	−1.13
					Post. cingulate cortex: decrease (*P* ≤ 0.0001)	−45 %	−1.63
					Dorsal pons: decrease	−8 %	−0.18
					Ventral pons: decrease	−22 %	−0.53
[¹²³I]β-CIT SPECT & phMRI following citalopram (SSRI)	Schouw et al. (2012)	*MDMA* 10 *PD-Controls* 7	*MDMA*: ecstasy >50 times *PD-Controls*: ecstasy-naive polydrug users	≥2-week abstinent of all drugs, urine screening	*SPECT*: *MDMA vs PD-Controls*:		
					Left anterior occipital lobe: decrease (*P* = 0.01)		^j^
					*phMRI + Citalopram (SSRI)*:		
					- Controls: no CBF change (*P* = 0.01)		
					- MDMA: significant (*P* = 0.01)CBF change in		
					- Thalamus: decrease and increase		
					- Right occipital cortex: decrease		
					- Right frontal cortex: decrease		
					- Left globus pallidus: increase		
					- Frontal cortex: increase		
					*MDMA vs PD-Controls*:		
					Mean whole-brain CBF: higher (*P* < 0.01)	12 %	0.57
[¹²³I]β-CIT SPECT	de Win et al. (2008a)^a^	*MDMA* 59 *PD-Controls* 56	*MDMA*: used ecstasy during follow up (prospective study) Average 6 tablets, median 2	≥2-week abstinent, urine screening	*MDMA vs PD-Controls*:		
					Midbrain: increase	4 %	0.16
					Thalamus: decrease	−1 %	−0.05
					Frontal grey matter: no change	0 %	0.11
					Occipital grey matter: decrease	−40 %	−0.21
					Temporal grey matter: decrease	−4 %	−0.08
[¹²³I]β-CIT SPECT	de Win et al. (2008b)	*Linear regression*: *Groups*: *MDMA-H* 33 *PD-Controls* 38 *Voxel-by-voxel analysis*: *Groups*: 1) *MDMA-H-PD* 10 2) *MDMA-cannabis* 4 3) *PD-Controls* 5 4) *Cannabis-Controls* 16 5) *DN-Controls* 12	*MDMA-H*: heavy users, >100 ecstasy tablets lifetime No use of other drugs *PD-Controls*: ecstasy-naive polydrug users, <10 ecstasy tablets lifetime Voxel-by-voxel analysis: *1) MDMA-H-PD*: heavy ecstasy polydrug users *2) MDMA-cannabis*: selective ecstasy and cannabis users *3) PD-Controls*: ecstasy-naive polydrug users *4) Cannabis-Controls*: ecstasy-naive cannabis users *5) DN-Controls*: drug-naive controls	≥2-week abstinent, urine screening, hair analysis	*Linear regression*:		
					*MDMA-H vs PD-Controls*:^b,c^		^j^
					Thalamus: decrease (*P* < 0.003)		
					Frontal cortex: decrease		
					Temporal cortex: decrease		
					*Voxel-by-voxel analysis*:		
					*MDMA vs PD-Controls*:^b,c^		
					Thalamus: decrease (*P* = 0.001)		
					Cingulate gyrus: decrease (*P* < 0.001) (Z-value exactly in midline)		
					Thalamus: decrease (*P* < 0.001) in		
					*- Ecstasy users vs substance using controls (g. 1,2 vs 3,4)*		
					*- Ecstasy polydrug users vs ecstasy-naive polydrug users (g. 1 vs 3)*		
					Anterior cingulate gyrus: decrease (*P* < 0.001) in		
					*- Ecstasy users vs substance using controls (g. 1,2 vs 3,4)*		
					Anterior cingulate gyrus: no decrease in		
					*- Ecstasy polydrug users vs ecstasy-naive polydrug users (g. 1 vs 3)*		
[¹²³I]β-CIT SPECT	Reneman et al. (2001a)^e,f^	*MDMA-M* 15 *MDMA-H* 23 *Ex-MDMA* 16 *PD-Controls* 15	*MDMA-M*: moderate use <50 tablets lifetime *MDMA-H*: heavy use >50 tablets lifetime *Ex-MDMA*: abstinent >12 months, >50 tablets lifetime *PD-Controls*: ecstasy-naive polydrug users	≥3-week abstinent, urine screening	Men^c,^ ^d^		
					*MDMA-M vs PD-Controls*:		^j^
					Overall binding ratio: decrease	−0.040	
					Midbrain: decrease	−0.073	
					Occipital cortex: decrease	−0.065	
					Thalamus: decrease	−0.026	
					*MDMA-H vs PD-Controls*:		
					Overall binding ratio: decrease	−0.025	
					Midbrain: decrease	−0.011	
					Occipital cortex: decrease	−0.032	
					Thalamus: decrease	−0.078	
					*Ex-MDMA vs PD-Controls*:		
					Overall binding ratio: increase	0.018	
					Midbrain: increase	0.019	
					Occipital cortex: decrease	−0.025	
					Thalamus: increase	0.095	
					*Ex-MDMA vs MDMA-H*:		
					Overall binding ratio: increase	0.04	
					Midbrain: increase	0.030	
					Occipital cortex: decrease	0.006	
					Thalamus: increase	0.174	
					*Women* ^c,d^		
					*MDMA-M vs PD-Controls*:		^j^
					Overall binding ratio: decrease	−0.058	
					Midbrain: decrease	−0.091	
					Occipital cortex: decrease	−0.082	
					Thalamus: decrease	−0.043	
					*MDMA-H vs PD-Controls*:		
					Overall binding ratio: decrease (*P* < 0.01)	−0.168	
					Midbrain: decrease (*P* = 0.013)	−0.154	
					Occipital cortex: decrease (*P* < 0.01)	−0.175	
					Thalamus: decrease	−0.221	
					*Ex-MDMA vs PD-Controls*:		
					Overall binding ratio: decrease	−0.031	
					Midbrain: decrease	−0.030	
					Occipital cortex: decrease	−0.075	
					Thalamus: increase	0.046	
					*Ex-MDMA vs MDMA-H*:		
					Overall binding ratio: increase (*P* = 0.004)	0.137	
					Midbrain: increase (*P* = 0.04)	0.124	
					Occipital cortex: increase (*P* = 0.02)	0.100	
					Thalamus: increase	0.268	
[¹²³I]β-CIT SPECT\	Reneman et al. (2001b)	*MDMA* 22 *Ex-MDMA* 16 *PD-Controls* 13	*MDMA*: >50 tablets lifetime *Ex-MDMA*: abstinent >12 months, >50 tablets lifetime	≥3-week abstinent, urine screening	*MDMA vs DN-Controls*:		
					Cortical: decrease (*P* < 0.03)*	−9 %	−1.08
					*Ex-MDMA vs DN-Controls*:		
					Cortical: decrease	−3 %	−0.35
[¹²³I]β-CIT SPECT	Semple et al. (1999)^g^	*MDMA* 10 *PD-Controls* 10	*MDMA*: > 50 tablets, >1 year, currently using on a regular basis	≥1-week abstinent, hair analysis, no formal check	*MDMA vs PD-Controls*:^c^		
					*Left side*:		
					Frontal: decrease	−4 %	−0.31
					Anterior cingulate: decrease	−5 %	−0.32
					Anterior temporal: decrease	−3 %	−0.21
					Middle temporal: decrease	−5 %	−0.38
					Occipital: decrease (*P* = 0.02)*	−10 %	−0.72
					Calcarine: decrease (*P* = 0.02)*	−13 %	−1.06
					Posterior cingulate: decrease	−7 %	−0.50
					Caudate: decrease	−3 %	−0.17
					Putamen: increase	4 %	0.26
					Thalamus: decrease	−2 %	−0.14
					Day 2 caudate: decrease	−8 %	−0.39
					Day 2 putamen: decrease	1 %	0.06
					*We excluded the results of the right side in this table.*		
[^11^C]McN5652 PET	Buchert et al. (2007)^h^	*MDMA* 30 *Ex-MDMA* 29 *PD-Controls* 28 *DN-Controls* 29	*MDMA*: regular ecstasy use >1 time a week or >2 tablets in 48 hours every time *Ex-MDMA*: lifetime intake 250–400 tablets Use >3 years, last use >20 weeks	≥3-day abstinent, urine screening	*MDMA vs Ex-MDMA*:		
					Mesencephalon: decrease (*P* ≤ 0.05)	−25 %	−0.87
					Putamen: decrease	−11 %	−0.38
					Caudate: decrease	−16 %	−0.53
					Thalamus: decrease (*P* ≤ 0.05)	−22 %	−0.72
					*MDMA vs DN-Controls*:		
					Mesencephalon: decrease (*P* ≤ 0.01)	−28 %	−1.20
					Putamen: decrease	−14 %	−0.58
					Caudate: decrease (*P* ≤ 0.05)	−18 %	−0.73
					Thalamus: decrease (*P* ≤ 0.01)	−24 %	−0.95
					*MDMA vs PD-Controls*:		
					Mesencephalon: decrease (*P* ≤ 0.001)	−32 %	−1.14
					Putamen: decrease (*P* ≤ 0.01)	−24 %	−0.96
					Caudate: decrease (*P* ≤ 0.01)	−21 %	−0.81
					Thalamus: decrease (*P* ≤ 0.01)	−26 %	−1.05
[^11^C]McN5652 PET	Thomasius et al. (2006)	*MDMA* 11 *Ex-MDMA* 10 *PD-Controls* 11 *DN-Controls* 15	*PD-MDMA*: ≥5 ecstasy tablets during follow up (2 years), polydrug pattern, heavy ecstasy use *Ex-MDMA-PD*: ≤5 tablets during follow up, former polydrug pattern, heavy ecstasy use *PD-Controls*: polydrug, ≤5 tablets ecstasy during follow up *DN-Controls*: drug-naive	≥6-day abstinent, urine screening, hair analysis	*MDMA vs DN-Controls*:^b^		
					Mesencephalon: decrease (*P* < 0.05)*	−7 %	−1.99
					Putamen: decrease	−6 %	−1.29
					Caudate nucleus: decrease	−7 %	−1.10
					Thalamus: decrease	−6 %	−1.29
					White matter: decrease	−7 %	−0.60
					*MDMA vs Ex-MDMA*:^b^		
					Mesencephalon: decrease (*P* < 0.05)*	−8 %	−1.96
					Putamen: decrease	−6 %	−0.99
					Caudate nucleus: decrease	−8 %	−1.17
					Thalamus: decrease	−7 %	−1.33
					White matter: decrease	−7 %	−0.60
					*MDMA vs PD-Controls*:^b^		
					Mesencephalon: decrease (*P* < 0.05)*	−7 %	−1.42
					Putamen: decrease	−6 %	−0.81
					Caudate nucleus: decrease	−7 %	−0.84
					Thalamus: decrease	−6 %	−0.87
					White matter: decrease	−2 %	−0.14
[^11^C]McN5652 PET	McCann et al. (2005)	*MDMA* 23 *PD-Controls* 19	*MDMA*: ecstasy >25 times on separate occasions *PD-Controls*: ecstasy-naive polydrug users	≥2-week abstinent, urine screening	*MDMA vs PD-Controls*:		
					Midbrain: decrease	−17 %	−0.43
					Amygdala: decrease (*P* ≤ 0.05)	−23 %	−0.63
					Hippocampus: decrease (*P* ≤ 0.01)	−39 %	−1.21
					Thalamus: decrease (*P* ≤ 0.01)	−30 %	−1.01
					Caudate: decrease (*P* ≤ 0.05)	−23 %	−0.73
					Putamen: decrease (*P* ≤ 0.05)	−20 %	−0.69
					DLPF cortex: decrease (*P* ≤ 0.01)	−52 %	−0.94
					Occipital cortex: decrease (*P* ≤ 0.01)	−54 %	−1.06
					Orbitofrontal cortex: decrease (*P* ≤ 0.05)	−32 %	−0.67
					Parietal cortex: decrease (*P* ≤ 0.01)	−42 %	−1.10
					Temporal cortex: decrease (*P* < 0.001)	−41 %	−1.30
					Ant. cingulate cortex: decrease (*P* ≤ 0.01)	−32 %	−1.10
					Post. cingulate cortex: decrease (*P* ≤ 0.01)	−35 %	−1.11
					Dorsal pons: decrease	−15 %	−0.40
					Ventral pons: decrease	−9 %	−0.17
[^11^C]McN5652 PET	McCann et al. (1998)	*MDMA* 14 *Controls* 15	*MDMA*: ecstasy >25 times on separate occasions *Controls*: ecstasy-naïve; however, use of other drugs is unclear	≥3-week abstinent, urine screening	*MDMA vs Controls*:^i^		
					Hypothalamus: decrease (*P* ≤ 0.05)	−31 %	^j^
					Midbrain: decrease (*P* ≤ 0.05)	−24 %	
					Thalamus: decrease	−34 %	
					Caudate: decrease (*P* ≤ 0.05)	−22 %	
					Putamen: decrease (*P* ≤ 0.05)	−25 %	
					Pons: decrease (*P* ≤ 0.05)	−25 %	
					Temporal cortex: decrease	−29 %	
					Cingulate cortex: decrease (*P* ≤ 0.05)	−29 %	
					Frontal cortex: decrease (*P* ≤ 0.05)	−28 %	
					Occipital cortex: decrease (*P* ≤ 0.05)	−26 %	
					Parietal cortex: decrease (*P* ≤ 0.05)	−24 %	
					Cerebellum: decrease (*P* ≤ 0.05)	−23 %	

This table reports the results of the human studies included on SERT. Only significant *P* values are shown. *P* values corrected for multiple comparisons are marked with the sign *, in studies in which a ROI or VOI analyses was performed. “MDMA” means MDMA users. “DN-Controls” are drug-naive controls. “MPU” is MDMA-preferring users (MDMA/hallucinogen number of lifetime exposures ratio >1). “HPU” is hallucinogen-preferring users (MDMA/hallucinogen number of lifetime exposures ratio <1). “Ex-MDMA” means former MDMA users. "PD-Controls" are polydrug users (excluding MDMA use). “MDMA-H” is heavy MDMA users. “MDMA-H-PD” is heavy ecstasy polydrug users. ”MDMA-cannabis” is selective ecstasy and cannabis users. “Cannabis-Controls” are ecstasy-naive cannabis users. “MDMA-M” is moderate MDMA users

^a^This study is a follow-up study (with repeated imaging) in part of the sample that was included by the study of De Win et al. (2008b)

^b^Additional longitudinal data not shown in the table

^*c*^Results of selection of brain regions

^d^Outcome presented in a logarithmic scale

^e^In this study, the study sample has been expanded as compared to Reneman and colleagues (2001b). Moreover, the study sample is identical to the sample of the study of de Win et al. (2004b)

_f_In this study, the data was not normally distributed and therefore expressed in a logarithmic scale. Consequently, the data could not be expressed in percentage change compared to control data

^g^There was no formal urine screening test to check the reported abstinence of drugs

^h^The study is a re-evaluation of Buchert et al. (2004)

^i^Estimated values, data was shown in a graphic

^j^ Not all results were shown in the publication; therefore, the effect sizes could not be calculated

#### 5-HT_2A_ receptor

As shown in Table 5, only five human studies examined in vivo the effects of MDMA on 5-HT_2A_ receptor binding. A couple of animal studies explored the effects of ecstasy administration on the 5-HT_2A_ receptor as well; however, those studies were excluded because they only used ex vivo imaging techniques. Out of these five human studies, three showed a significant increase in 5-HT_2A_ receptor binding in MDMA users compared to controls. In contrast, the other two studies showed a significant decrease of binding.

**Table 5:** 5-HT_2A_ human studies

Technique used	Author	Nr pts/controls	Inclusion and exclusion criteria	MDMA check	Mean time of abstinence	Outcome		Effect size
[¹¹C] MDL 100907 PET	Urban et al. (2012)	*MDMA* 13 *DN-Controls* 13	*MDMA*: ecstasy >15 times, regularly 12 months before enrolment Occasional recreational use of other substances was permitted *DN-Controls*: drug-naive	≥2-week abstinent, urine screening, hair analysis	5.7 weeks (2–8 weeks)	*MDMA vs DN-Controls*:		
						Dorsolateral prefrontal cortex: increase (*P* = 0.04)	16 %	0.86
						Medial prefrontal cortex: increase	2 %	0.14
						Orbitofrontal cortex: increase	14 %	0.62
						Temporal cortex: increase	10 %	0.52
						Medial temporal lobe: increase	8 %	0.32
						Parietal cortex: increase (*P* = 0.03)	19 %	0.87
						Occipital cortex: increase	13 %	0.71
						Anterior cingulate: increase	8 %	0.46
						Insula: increase	12 %	0.64
						Amygdala: decrease	−1 %	−0.05
						Entorhinal cortex	−10 %	−0.31
						Hippocampus: increase	15 %	0.59
						Uncus: increase	8 %	0.21
						Caudate: increase	100 %	0.45
						Putamen: increase	71 %	0.53
						Thalamus: increase	11 %	0.14
						Midbrain: decrease	−75 %	0.74
[^18^F] Setoperone PET	Di Iorio et al. (2012)	*MDMA* 14 *PD-Controls* 10	All Female *MDMA*: ecstasy >5 times *PD-Controls*: ecstasy-naive polydrug users *Both groups*: cocaine, lysergic acid diethylamide and other amphetamines >90 days prior to study	≥2-week abstinent, urine screening, hair analysis	98.5 weeks (34.0–169.8 weeks)	*MDMA vs PD-Controls*:		
						Occipital-parietal: increase (*P* = 0.001)	20 %	1.69
						Temporal: increase (*P* = 0.003)	20 %	1.42
						Occipitotemporal-parietal: increase (*P* ≤ 0.001)	18 %	1.84
						Frontal: increase (*P* = 0.002)	17 %	1.53
						Frontoparietal: increase (*P* = 0.004)	18 %	1.36
						No regions in which serotonin_2A_ BP_ND_ was lower in MDMA users than in controls (*P* > 0.05)		^b^
[^18^F] Altanserin PET	Erritzoe et al. (2011)	*MDMA-preferring users (MPU)* 14 *Hallucinogen-preferring users (HPU)*: 10 *DN-Controls*: 21	*MPU or HPU*: ecstasy or hallucinogens >12 times *MPU*: MDMA/hallucinogen number of lifetime exposures ratio >1 *HPU*: MDMA/hallucinogen number of lifetime exposures ratio <1 *DN-Controls*: drug-naive Cannabis up to 15 exposures	≥1-week abstinent, urine screening, hair analysis	8.1 weeks (1.6–36.9 weeks)	*MPU/HPU vs DN-Controls*:		
						Neocortex: decrease (*P* = 0.03)*	−9 %	^b^
						Orbitofrontal cortex: decrease	−13 %	
						Medial inferior frontal cortex: decrease	−10 %	
						Superior frontal cortex: decrease	−7 %	
						Superior temporal cortex: decrease	−11 %	
						Medial inferior temporal cortex: decrease	−13 %	
						Sensory motor cortex: decrease	−7 %	
						Parietal cortex: decrease	−8 %	
						Occipital cortex: decrease	−4 %	
[^123^I] 5-I-R91150 SPECT	Reneman et al. (2002c)	*MDMA*: 17 *Ex-MDMA* 7 *PD-Controls* 11	*MDMA*: >50 tablets *Ex-MDMA*: >50 tablets, >2 months drug free *PD-Controls*: ecstasy-naive polydrug users All groups were permitted to use other substances	≥1-week abstinent, urine screening	*MDMA*: 3.3 weeks *Ex-MDMA*: 19.6 weeks	*MDMA vs PD-Controls*:^a^		
						All brain regions studied: decrease		
						Frontal cortex: decrease (*P* < 0.01)*	−19 %	
						Parietal cortex: decrease (*P* < 0.01)*	−19 %	
						Occipital cortex: decrease (*P* = 0.04)*	−12 %	
						*Ex-MDMA vs MDMA*:^a^		
						Frontal cortex: increase	30 %	
						Parietal cortex: increase	31 %	
						Occipital cortex: increase	32 %	
						*Ex-MDMA vs PD-Controls*:^a^		
						Frontal cortex: increase	6 %	
						Parietal cortex: increase	5 %	
						Occipital cortex: increase (*P* = 0.04)*	16 %	
[^123^I] 5-I-R91150 SPECT	Reneman et al. (2000)	*MDMA* 5 *DN-Controls* 9	*MDMA*: abstinent users, lifetime intake 50–500 tablets *DN-Controls*: drug-naive	≥2-month abstinent, urine screening	19.9 weeks (8.7–47.7 weeks)	*MDMA vs DN-Controls*:		
						Overall: increase		^b^
						Occipital cortex: increase (*P* < 0.05)	17 %	1.54

This table reports the results of the human studies on the 5-HT_2A_ receptor. Only significant *P* values are shown. *P* values corrected for multiple comparisons are marked with the sign *, in studies in which a ROI or VOI analyses was performed. “MDMA” means MDMA users. “DN-Controls” are drug-naive controls. “PD-Controls” are polydrug controls (excluding MDMA use). “MPU” is MDMA-preferring users (MDMA/hallucinogen number of lifetime exposures ratio >1). “HPU” is hallucinogen-preferring users (MDMA/hallucinogen number of lifetime exposures ratio <1). “Ex-MDMA” means former MDMA users

^a^Estimated values, data was shown in a graph

^b^Not all results were shown in the publication; therefore, the effect sizes could not be calculated

#### 5-HT_1A_ receptor

In this review, we found one animal study on the 5-HT_1A_ receptor, which could be included. As can be seen from the data presented in Table 6, no significant differences in 5-HT_1A_ receptor binding were found between the baseline scan and the scan after MDMA treatment.

**Table 6:** 5-HT_1A_ receptor animal studies

Technique used	Author	Nr pts/controls	Details animals	Dosage drugs	Outcome		Effect size
[^11^C]WAY-100635 PET	Cumming et al. (2007)	*Baseline + MDMA* 4	Göttingen minipigs	*MDMA*: Mean 42 mg/kg	No consistent or significant effect of MDMA treatment on [^11^C]WAY-100635 in any brain region		^a^

This table shows the results of the animal studies included into the 5-HT_1A_ receptor. Only significant *P* values (not corrected for multiple comparisons) are presented. “Baseline + MDMA” means that a baseline scan was taken, followed by that MDMA was given and a second scan was taken

^a^Not all results were shown in the publication; therefore, the effect sizes could not be calculated

#### Serotonergic vesicular monoamine transporter 

The vesicular monoamine transporter (VMAT) is expressed in all monoaminergic neurons. However, in 5-HT-rich brain areas, such as the hypothalamus, VMAT expression represents preferentially VMAT expression in 5-HT neurons. Solely, Fantegrossi et al. (2004) studied VMAT binding in 5-HT-rich parts of the brain (midline structures consisting of thalamic and hypothalamic nuclei) as is shown in Table 7. Seven monkeys were studied, whereof four monkeys self-administered MDMA. No significant differences in VMAT binding were reported between both two groups.

**Table 7:** 5-HT-ergic vesicular monoamine transporter (VMAT) animal studies

Technique used	Author	Nr pts/controls	Details animals	Dosage drugs	Outcome		Effect size
[^11^C]DTBZ PET	Fantegrossi et al. (2004)	*SA-MDMA* 4 *PD-Controls* 3	Rhesus monkeys	Extensive drug self-administration including opioids and psychostimulants *MDMA* 1190–2508 mg *PD-Controls*: MDMA and METH-naive	*SA-MDMA vs PD-Controls*:		
					DVR midline structures (thalamic and hypothalamic nuclei) *vs* occipital cortex: no significant differences^a^		^b^

This table reports the results of the animal studies included on 5-HT-ergic VMAT. “SA-MDMA” is monkeys that self-administered MDMA. “PD-Controls” are polydrug controls (excluding MDMA self-administration). “DVR” is the distribution volume ratio of [^11^C]DTBZ

^a^Also presented in Table 11: DA-ergic VMAT

^b^Not all results were shown in the paper; therefore, the effect sizes could not be calculated

### Dopamine system

#### Dopamine D_2/3_ receptor and dopamine release

One study was included that explored the effect of MDMA on striatal DA D_2/3_ receptors and endogenous DA release (Table 8). At baseline level, striatal D_2/3_ binding was lower in ex-MDMA users than controls, in all subdivisions of the striatum, although this result was not statistically significant. After playing a video game, ex-MDMA users seemed to have a lower DA release in both left and right caudate nucleus and putamen than controls. However, none of these differences were statistically significant and ES were relatively low ranging from 0.07 to 0.32.

**Table 8:** DA D_2/3_ + DA release human studies

Technique used	Author	Nr pts/controls	Inclusion and exclusion criteria	MDMA check	Outcome		Effect size
[^123^I]IBZM SPECT	Weinstein (2010)^**a**^	*Ex-MDMA* 9 *PD-Controls* 8	Ex-MDMA: mean 5-month abstinent (1–18 months) Use of other drugs PD-Controls: no current or recent use of ecstasy or marijuana	1–18-month abstinent, no formal urine screening test	*Ex-MDMA*:^b^		
					*After videogame vs baseline*:		
					Left caudate: decrease	−6 %	0.07
					Right caudate: decrease	−3 %	0.07
					Left putamen: decrease	−1 %	0.10
					Right putamen: decrease	1 %	0.10
					*PD-Controls*:^b^		
					*After videogame vs baseline*:		
					Left caudate: decrease	−17 %	0.15
					Right caudate: decrease (*P* < 0.05)	−13 %	0.21
					Left putamen: decrease	−7 %	0.29
					Right putamen: decrease	−9 %	0.32
					*Baseline*:		
					*Ex-MDMA vs PD-Controls*:		
					Left caudate: decrease	−17 %	−0.41
					Right caudate: decrease	−19 %	−0.50
					Left putamen: decrease	−16 %	−0.44
					Right putamen: decrease	−17 %	−0.53

This table shows the results of human studies into DA D_2/3_ + DA release. Only significant *P* values (not corrected for multiple comparisons) are presented. “Ex-MDMA” is former MDMA users. “PD-Controls” are polydrug controls (excluding MDMA use)

^a^Included patients that used antipsychotic treatment; however, they did not take their medication for 6 months at the time of the scans. There was no formal urine screening test to check the reported abstinence of drugs

^b^More longitudinal data not shown in the table

#### Dopamine transporter

Three studies were found and included that examined the DAT in ecstasy users (Table 9). One study showed a significant increase of 13 % in striatal binding ratios of MDMA users compared to controls (striatal binding ratios, *P* = 0.045, ES = 2.92), whereas the other two studies did not show any significant difference.

**Table 9:** DAT human studies

Technique used	Author	Nr pts/controls	Inclusion and exclusion criteria	MDMA check	Outcome		Effect size
[^11^C]WIN 35,428 PET	McCann et al. (2008)	*MDMA* 16 *PD-Controls* 16	*MDMA*: ecstasy >25 times, two or more doses over a 3–12-h period	≥2-week abstinent, urine screening	*MDMA vs PD-Controls*:		^a^
					Caudate: no difference		
					Putamen: no difference		
[¹²³I]β-CIT SPECT	Reneman et al. (2002b)	*MDMA* 29 *MDMA + AMPH* 9 *PD-Controls* 15	*MDMA + AMPH*: used amphetamines <3 weeks before study *PD-Controls*: ecstasy-naive polydrug users	≥3-week abstinent, urine screening	*MDMA vs PD-Controls*:		
					Striatal binding ratios: increase (*P* = 0.045)	13 %	2.92
					*MDMA + AMPH vs MDMA*:		
					Striatal binding ratios: decrease (*P* = 0.007)	−20 %	−4.09
					*MDMA + AMPH vs PD-Controls*:		
					Striatal binding ratios: decrease	−10 %	−1.57
[¹²³I]β-CIT SPECT	Reneman et al. (2001a)	*MDMA-H* 23 *PD-Controls* 15	*MDMA-H*: heavy use >50 tablets lifetime *PD-Controls*: ecstasy-naive polydrug users	≥3-week abstinent, urine screening	*MDMA-H vs PD-Controls*:		^a^
					Striatal binding ratios: no significant differences		

This table reports the results of the human studies included on DAT. Only significant *P* values (not corrected for multiple comparisons) are presented. “MDMA” means MDMA users. “PD-Controls” are polydrug controls (excluding MDMA use). “MDMA + AMPH” is MDMA users that also use amphetamines

^a^Not all results were shown in the publication; therefore, the effect sizes could not be calculated

#### Decarboxylase activity ([^*18*^*F*]dopa positron emission tomography)

Table 10 presents data of one study that was included examining decarboxylase activity. This research indicated that decarboxylase activity was increased in the caudate nucleus, putamen (putamen, *P* = 0.021, ES = 1.10) and ventral striatum comparing ex-MDMA users to drug-naive controls. ES ranged from 0.52 to 1.10. Ex-MDMA users were also compared to polydrug using controls, but this comparison showed no significant effect anymore (ES ranged from −0.04 to 0.47).

**Table 10:** Decarboxylase activity human studies

Technique used	Author	Nr pts/controls	Inclusion and exclusion criteria	MDMA check	Outcome		Effect size
[^18^F]dopa PET	Tai et al. (2011)	*Ex-MDMA* 14 *PD-Controls* 14 *DN-Controls* 12	*Ex-MDMA*: abstinent >1 year, ecstasy >25 times, allowed to continue other recreational drugs *PD-Controls*: ecstasy-naive polydrug users *DN-Controls*: drug-naive except alcohol	≥3-day abstinent, urine screening, hair analysis	*Ex-MDMA vs DN-Controls*:		
					Caudate: increase	5 %	0.52
					Putamen: increase (*P* = 0.021)*	9 %	1.10
					Ventral striatum: increase	6 %	0.68
					*Ex-MDMA vs PD-Controls*:		
					Caudate: no difference	0 %	−0.04
					Putamen: increase	4 %	0.46
					Ventral striatum: increase	4 %	0.47
					*PD-Controls vs DN-Controls*:		
					Caudate: increase	5 %	0.55
					Putamen: increase	5 %	0.73
					Ventral striatum: increase	2 %	0.26

This table reports the results of human studies into decarboxylase activity. Only significant P-values are shown. P-values corrected for multiple comparisons are marked with the sign *. “Ex-MDMA” means former MDMA users. “DN-Controls” are drug-naive controls. “PD-Controls” are polydrug controls (excluding MDMA use)

#### Vesicular monoamine transporter-dopaminergic

In this review, one animal study was found that investigated the VMAT in a DA-ergic brain area (basal ganglia) (Table 11). No significant differences were found in distribution volume ratios comparing MDMA self-administering monkeys to drug-naive controls.

**Table 11:** DA-ergic vesicular monoamine transporter (VMAT) animal studies

Technique used	Author	Nr pts/controls	Details animals	Dosage drugs	Outcome		Effect size
[^11^C]DTBZ PET	Fantegrossi et al. (2004)	*SA-MDMA* 4 *PD-Controls* 3	Rhesus monkeys	Extensive drug self-administration including opioids and psychostimulants *MDMA* 1190–2508 mg *PD-Controls*: MDMA and METH-naive	*SA-MDMA vs PD-Controls*:		^b^
					DVR of basal ganglia *vs* occipital cortex: no significant differences^a^		

This table reports the results of the animal studies included on DA-ergic VMAT. “SA-MDMA” is monkeys that self-administered MDMA. “PD-Controls” are polydrug controls (excluding MDMA self-administration). “DVR” is the distribution volume ratio of [^11^C]DTBZ

^a^Also presented in Table 7: 5-HT-ergic VMAT

^b^Not all results were shown in the publication; therefore, the effect sizes could not be calculated

## Discussion

Results of molecular imaging studies showed quite consistently that SERT binding is lower after use/administration of ecstasy/MDMA, particularly after administration of high dosages, while studies on the 5-HT_2A_ receptor showed inconsistent results. Results of molecular imaging studies on the DA system are quite consistent in that most molecular imaging studies in humans did not find any significant effect of MDMA on the dopamine system. Here, we will focus primarily on the statistically significant findings reported in Tables 2, 3, 4, 5, 6, 7, 8, 9, 10 and 11.

### 5-Hydroxytryptamine synthesis

In this review, only one human study on 5-HT synthesis was included (Table 2). The main reason that, until recently, only one study looked into 5-HT synthesis in MDMA users is that the PET radiotracer, alpha-[^11^C]-methyl-l-tryptophan ([^11^C]AMT), which is a well-validated radiotracer to measure 5-HT synthesis, is hardly available. In this study, only 17 MDMA users and 18 age-matched controls were included, whereof half of the MDMA users and controls were men. Increases and decreases in [^11^C]AMT trapping were observed comparing MDMA users with controls; however, decreases were mainly seen in prefrontal–orbital and parietal regions and increases in the brainstem. The differences were more extensive in men than in women. As suggested by the authors, the decreases in the forebrain may reflect 5-HT neurotoxicity and the increases in the brainstem could be explained by an up-regulation of synthesis to compensate the loss of 5-HT neurons. Nevertheless, further research should be performed to draw definitive conclusions whether 5-HT synthesis is altered in MDMA users. Also, it may be relevant to perform studies in small laboratory animals with this radiotracer, to validate whether administration of MDMA is able to induce detectable changes in 5-HT synthesis as assessed by this radiotracer and to study the relationship between 5-HT synthesis and 5-HT neurotoxicity.

### Serotonin transporter

Eleven animal studies looked into the effects of MDMA on SERT binding, and all of them showed lower SERT binding, reaching statistical significant effects in ten of these studies (Table 3). The ES were large (ranging from −0.38 to −20.03), which indicates that the effect of MDMA on SERT binding is a robust finding in animals. As compared to human studies, an advantage of animal studies is that the animals were solely treated with MDMA. In humans, however, polydrug use is common, which makes it harder to look at the effects of MDMA per se (Gouzoulis-Mayfrank and Daumann 2006). Consequently, it may be hard to generalize the findings observed in animals to humans. Also, in animal studies, MDMA was administered frequently. Indeed, most of the animal studies administered MDMA twice a day for 4 days in a row, whereas humans typically only use one or two tablets of ecstasy in the weekend. Moreover, relatively high doses within a short interval (e.g. two doses per day for four consecutive days) of MDMA were used in the animal studies (range 20–141 mg/kg), which may explain the large ES, and the drug was administered commonly intraperitoneally. However, some research indicates that due to differences in metabolism, neurotoxic dosages of MDMA are different between small animal species and primates. In rats, only high dosages of at least 20 mg/kg may be neurotoxic (Schmidt 1987). Using differences in clearance and body mass/surface area between monkeys and humans, an estimation of the neurotoxic dosage of MDMA for a human can be made, which was estimated at 1.28 mg/kg by Ricaurte et al. (2000). As mentioned before, humans typically use one or two tablets of ecstasy, each containing approximately 138 mg (reflecting 2–4 mg/kg in a person of 70 kg) (Van Laar et al. 2015). This dosage may be in the neurotoxic range based on the prediction by Ricaurte et al. (2000). In contrast, Baumann et al. (2007) argued that interspecies scaling, which means adjusting doses between species, should not be used, because behavioural, endocrine and neurochemical reactions will occur at corresponding doses, around 1–2 mg/kg. Furthermore, other researchers argued that high doses, i.e. >25 mg/kg, of MDMA produce neurotoxicity to all types of neurons (Jensen et al. 1993). These findings implicate that the doses of MDMA used in most animal research might be too high to compare the results of these studies with human studies.

Consistent with findings in animals, 14 out of the 16 SERT studies performed in humans also showed significantly lower SERT binding, particularly in cortical brain areas. However, not all studies corrected for multiple comparisons. Ten studies examined SERT binding in the occipital cortex, and in six of these studies, the decrease of SERT binding was most pronounced in this particular brain area, with ES ranging from −0.21 to −2.17. Several experimental studies have reported that, indeed, high doses of ecstasy affect preferentially 5-HT-ergic projections to the occipital cortex (Oliveri and Calvo 2003). Hadzidimitrou and colleagues (1999) and Molliver et al. (1990) stated that axons with a great length, e.g. axons to the occipital cortex, have a higher sensitivity to neurotoxic substances. Besides the cortical regions, forebrain regions were also examined. Four studies explored hippocampal SERT, and in 3 out of these 4 studies was the SERT binding significantly reduced in MDMA users; however, only 2 were corrected for multiple comparisons. It has been shown that heavy MDMA users have verbal and visuo-spatial memory deficits, and loss of SERT in the hippocampus may contribute to these deficits (Bosch et al. 2013). For cognitive processes like language and memory, the thalamus is also very important (Herrero et al. 2002). Eleven studies found that the SERT binding was lower in this brain area in users with a history of ecstasy use; however, only three studies showed significant effects. It might be that SERT loss in the thalamus plays a key role in verbal memory deficits too.

The study of Urban et al. (2012) showed statistically non-significant decreases of −100 % in SERT binding in the orbitofrontal and parietal cortex. These large percentages can be explained by the fact that the binding of [¹¹C]DASB in these regions is very low, which hampers an accurate quantification of SERT binding.

It should be considered that different radiotracers with different binding characteristics were used in studies on the effects of MDMA use/administration on SERT, which may have influenced outcomes. SPECT studies used the non-selective tracer [^123^I]β-CIT, while PET studies used selective tracers, e.g. [^11^C]DASB and [^18^F]ADAM (Chen et al. 2012; Frankle et al. 2004). Since [^123^I]β-CIT binds with high affinity to both the DAT and SERT, SERT binding in DAT-rich areas (i.e. striatum) cannot be assessed with this radiotracer. Other methodological issues could have affected the accuracy of the quantitative measurements as well. For example, simple ratio methods were used in the SERT SPECT studies, which are more prone to changes in tracer delivery, whereas modelling time activity curves were used in some PET studies (e.g. the study of Booij et al. (2014)). Finally, the limited spatial resolution of PET scanners, and particularly of clinical SPECT scanners, can lead to an underestimation of the binding potential in small volumes (partial volume effect) (Erlandsson et al. 2012).

In the past 10 years, another technique called pharmacological MRI was evaluated to assess 5-HT dysfunction. This technique measures the hemodynamic response on a pharmaceutical, e.g. a selective serotonin reuptake inhibitor (SSRI). It is a very interesting development; however, more research is necessary to validate this technique (Schouw et al. 2012; Wingen et al. 2008).

Although the results of the included studies may be influenced by differences in tracer and techniques (PET versus SPECT, but also analysis techniques), the findings of imaging studies on SERT were robust. Confirming previous studies, use/administration of MDMA declines SERT binding.

### 5-HT_2A_ receptor

Three out of five imaging studies showed an increased 5-HT_2A_ binding in MDMA users (Table 5). In these three studies, the period of abstinence for ecstasy ranged between 2.0 and 169.8 weeks and ES ranged between 0.14 and 1.84. The other two studies showed a loss of 5-HT_2A_ receptor binding, and in these studies, the period of abstinence ranged between 1.6 and 36.9 weeks. The [^123^I]R91150 SPECT study of Reneman et al. (2002c) showed that in recent MDMA users (mean time of abstinence 3.3 weeks), postsynaptic 5-HT_2A_ receptor binding was significantly lower in all cortical areas studied, while 5-HT_2A_ receptor densities were significantly higher in the occipital cortex of ex-MDMA users. Moreover, this study showed a significant positive correlation between cortical 5-HT_2A_ receptor binding and duration of abstinence from MDMA (*P* < 0.01). Also, the same study showed, using an ex vivo technique in rats and using the same radiotracer, a decrease of binding followed by a time-dependent recovery of cortical 5-HT_2A_ receptor binding, which was strongly and positively associated with the degree of 5-HT depletion (Reneman et al. 2002c). However, no positive correlation between the 5-HT_2A_ receptor binding and time of abstinence was found in the other studies (Di Iorio et al. 2012; Erritzoe et al. 2011; Urban et al. 2012). The time of abstinence in the study of Erritzoe et al. (2011) ranged between 1.6 and 36.9 weeks and in the study of Di Iorio et al. (2012) between 34 and 169.8 weeks, so these ranges should be large enough to evaluate a possible correlation between 5-HT_2A_ receptor binding and time of abstinence. Moreover, the study of Urban et al. (2012) did not show a decrease in receptor binding, although the subjects were also relatively recent MDMA users (mean time of abstinence 5.7 weeks, ranging from 2 to 8 weeks), comparable to the study of Reneman et al. (2002c). So, all in all, findings on 5-HT_2A_ receptor binding in MDMA users are inconsistent and it is uncertain if there is a relationship between time of abstinence and 5-HT_2A_ receptor binding.

### Dopamine system (dopaminergic vesicular monoamine transporter, D_2/3_ receptor and dopamine release, dopamine transporter, decarboxylase activity)

Some experimental studies in animals suggested that administration of MDMA/ecstasy affects not only the 5-HT system, but also the DA system. For example, Commins et al. (1987) showed that when MDMA was given to rats in a high dosage, DA levels were decreased in some brain regions. However, other research showed that treatment with MDMA/ecstasy has limited effect on the dopamine nerve endings in rats (Battaglia et al. 1987; Stone et al. 1986). In mice, MDMA seems to be a selective DA neurotoxin, while in rats a selective 5-HT neurotoxin (Stone et al. 1986). Therefore, Easton and Marsden questioned the ability to translate findings of animal studies on DA neurotoxicity to humans (Easton and Marsden 2006).

In this search, we found one animal study and five human imaging studies that examined the influence of ecstasy on the central DA system and they showed consistently no significant effects of MDMA on the DA system (Tables 8, 9, 10 and 11). One study in monkeys examined the VMAT expression in the basal ganglia but did not find significant differences between the self-administering MDMA group and the polydrug-administering control group. One human study explored the effect of MDMA on baseline DA D_2/3_ receptors and DA release and no significant differences were found. Three studies examined striatal DAT binding in MDMA users; however, only one study of Reneman et al. (2002b) showed statistically significant differences. In that particular study, the effects of use of MDMA and amphetamines on striatal DAT binding were assessed. MDMA users were compared to polydrug using controls and the binding ratios in the striatum were significantly increased (striatal binding ratios, increase 13 %, *P* = 0.045, ES = 2.92). However, comparing MDMA users that used amphetamines less than 3 weeks before the study to MDMA users, it was found that striatal binding ratios were significantly decreased (striatal binding ratios, decrease 20 %, *P* = 0.007, ES = −4.09). This study concluded that use of amphetamines, and not the use of MDMA, might induce loss of nigrostriatal DA neurons. Because of the polydrug use of many ecstasy users, it is hard to look specifically at the effects of MDMA and they stressed the importance of the inclusion of a proper control group.

Only one study (Tai et al. 2011) looked into decarboxylase activity (using [^18^F]dopa PET) and found that there was a significant increase in ex-MDMA users compared to drug-naive controls, only in the putamen. However, the ex-MDMA users were polydrug users and when comparing ex-MDMA users to polydrug using controls, there was no significant effect anymore. This study stresses the importance of a well-selected control group as well.

In short, the results on the DA system are quite consistent. Most molecular imaging studies in humans did not find any significant effect of MDMA on the DA system. Further research has to be conducted to draw definite conclusions whether this system is affected in MDMA users.

### Limitations

Several limitations of this review should be recognized. In this review, we did not find imaging studies that assessed other neurotransmitter systems than the 5-HT or DA system that might be affected by MDMA. There is little imaging research available on other receptors/transporters that may be influenced by MDMA due to a lack of well-validated radiotracers for every transporter/receptor of interest. Moreover, most of the included studies used a very small number of subjects; the number of subjects in animal studies was ranging from 1 to 26 animals and in human studies from 14 to 116 subjects. Another limitation is the washout period used. A reasonable period of abstinence of MDMA/ecstasy is necessary to exclude direct pharmacological effects of MDMA on the neurotransmitter systems; this is of particular importance in studies on the 5-HT and DA system. However, some studies in this review used a minimal period of abstinence for ecstasy of only 1 week. Furthermore, the purity of ecstasy tablets varies and the amount of MDMA in a tablet changed over the years; consequently, there are limitations in comparing the results of the human studies over time (Sherlock et al. 1999). Also, not all studies were corrected for multiple comparisons, and therefore, some significant findings could be explained by chance.

### 3,4-Methylenedioxymethamphetamine and additional drug use

MDMA users are likely to be polydrug users. Several studies attempted to look specifically at the effects of MDMA by including polydrug using control groups. The study of Tai et al. (2011) (Table 10) showed the importance of a polydrug using control group, because there was no significant difference left in decarboxylase activity when the data of the MDMA group were compared to the data obtained in the polydrug control group. Different subgroups of polydrug users were also analysed by two studies to investigate the effects of some commonly used drugs in combination with MDMA, e.g. cannabis, cocaine and hallucinogens, on the binding of several transporters/receptors. This approach can be useful, because it may assess the influence of those drugs on the outcome of studies that included drug-naive controls instead of polydrug controls.

First, the study of de Win et al. (2008b) assessed the specific/independent neurotoxic effects of heavy ecstasy use and contributions of amphetamine, cocaine and cannabis. They concluded that use of cannabis and cocaine did not have any significant effect on the effects of MDMA on SERT binding as measured with [^123^I]β-CIT SPECT, comparing MDMA users with polydrug using controls. In the second study of Erritzoe et al. (2011), reductions were seen in the cerebral SERT binding in MDMA-preferring users, but not in hallucinogen-preferring users, and they concluded that not hallucinogens, but MDMA alters the presynaptic 5-HT-ergic transmitter system. Taken these studies into account, use of cannabis, cocaine and hallucinogens may not influence the effects of MDMA on the SERT significantly.

### Age-of-first exposure

One study (Klomp et al. 2012) looked into the effects of age-of-first exposure on SERT binding in humans and rats. In the early-exposed group, they found a significant inverse relationship between age-at-first ecstasy use and [^123^ I]β-CIT binding ratios in the SERT-rich midbrain; however, in the late-exposed group, no significant relationship was seen. They stated that, particularly, the developing brain might be sensitive to the potential neurotoxic effects of MDMA use. In early-exposed rats and humans however, they did not find lower SERT binding ratios in the midbrain. A likely explanation may be that the midbrain of rats is already matured very early in the maturation process; consequently, the effects of MDMA are less pronounced. These results suggest that in future studies, age-of-first exposure should be taken into account. Animal studies already concluded that the maturing brain is affected differently by the administration of MDMA/ecstasy (Broening et al. 1994; Meyer and Ali 2002); however, no animal studies on this topic were included. Only one human in vivo imaging study passed our inclusion criteria; therefore, more research has to be done to draw valid conclusions about what the role of age-of-first exposure is on changes to neurotransmitter systems in humans.

### Gender differences

Reneman et al. (2001a) reported about gender differences in susceptibility to possible neurotoxic effects of MDMA use. Several studies looked into this topic and came to different conclusions. Buchert et al. (2004) confirmed the association between sex and reduction of SERT availability. However, de Win et al. (2008b) did not find a gender effect on SERT availability. The only study that looked into 5-HT synthesis reported on a decreased [^11^C]AMT trapping in frontal regions in males, but not in women (Booij et al. 2014). In this study, men seemed to be more susceptible to the effects of polydrug use. In conclusion, whether gender plays an important role in susceptibility to the effects of MDMA use is not completely clear and further research on this topic should be undertaken.

### Alteration in receptor binding and neurotoxicity

The main outcome of imaging research is commonly expressed in terms of increased or decreased receptor/transporter binding; however, the cause of the alteration remains unclear in these studies. There are at least four explanations for the observed decrease in receptor/transporter binding: down-regulation and/or endocytosis of the receptor/transporter, neuronal damage resulting in loss of receptors/transporters which are expressed on this particular neuron, decreased expression of protein levels of the receptor and endogenous neurotransmitter release induced by the drug which could reduce the binding of the radiotracer (e.g. administration of MDMA/ecstasy can induce 5-HT release, which can lead to lower 5-HT_2A_ receptor availability). In this regard, it is of interest that a study by Quelch et al. (2012) showed a significant reduction in the ability of the radioligand [^3^H]DASB to bind to the SERTs that are located intracellularly (as compared to binding on the SERT expressed on the cell membrane) and they speculate that down-regulation could (partly) explain the reductions in SERT binding in MDMA studies with the radioligand [^11^C]DASB, since MDMA has been shown to redistribute SERT into intracellular compartments (Kivell et al. 2010). To distinguish between causes of lower receptor/transporter binding, further research in animal brains, e.g. using electron microscopy (to assess internalization of receptor binding) or high-performance liquid chromatography to assess neurotransmitter concentrations and determination of *B*_max_ (number of binding sites) and *K*_d_ (affinity for the receptor), would be helpful.

Also, more translational research is necessary to examine in which conditions lower SERT binding may reflect neurotoxicity. This is relevant since it is still debated whether ecstasy use/administration is indeed neurotoxic. There are several techniques developed that claim to measure neurotoxicity, e.g. immunocytochemistry, immunohistochemistry, reactive gliosis and silver staining. However, these techniques differ in sensitivity and specificity and it can be questioned whether they all can demonstrate 5-HT neurotoxicity. Immunocytochemistry can be used to look at the structural and functional integrity of the assessed neurotransmitter system. Immunohistochemistry can be used to assess 5-HT axon degeneration; with this technique concentrations of 5-hydroxyindoleacetic acid (5-HIAA), 5-HT and the SERT can be measured (Battaglia et al. 1987; Commins et al. 1987). Glial activation (reactive gliosis) is a response to all nervous system injury, and silver staining is a direct way to stain degenerating neurons (O’Callaghan and Sriram 2005). However, there are several limitations of these techniques. It is argued that immunohistochemistry should be validated by other means, because the neurotransmitter levels could be unmeasurable due to pharmacological depletions, while the neuron itself can be intact (Baumann et al. 2007; Chang and Slikker Jr 1995). Silver staining is not selective for damage to serotonergic axons but also measures loss of other types of neurons. However, this technique is very useful for measuring neuronal loss (Jensen et al. 1993; O’Callaghan and Miller 1993). In fact, no SPECT or PET tracer is available that can directly assess serotonergic/dopaminergic toxicity or degeneration per se. So, until yet, whether or not a lowered receptor/transporter binding as assessed by PET/SPECT studies in humans represents neurotoxicity is still a matter of interpretation. Importantly, MDMA is not only used recreationally. Some researchers proposed to treat patients with MDMA, e.g. as a catalyst in psychotherapy for PTSD patients (Amoroso 2015; Doblin et al. 2014; White 2014), which further highlights the need to be able to assess whether or not the use of ecstasy is neurotoxic to humans.

### Recovery

Not only the causes, but also the duration of the effects of MDMA/ecstasy on receptor/transporter binding is important to be further explored. As mentioned earlier, a number of studies have investigated the effect of the duration of ecstasy abstinence on the SERT binding by examining the reversibility of the SERT binding in relation to period of abstinence from MDMA use/administration. Scheffel et al. (1998) already showed in a baboon study that SERT binding was increased from 40 days to 9 months after MDMA administration in the pons, midbrain and hypothalamus, whereas it remained decreased in cortical regions (pons: increase 35.7 %, midbrain: increase 95 %, hypothalamus: increase 168.5 %). In human studies, similar results were found. Reneman et al. (2001b) concluded that SERT binding of ex-MDMA users that stopped using MDMA for more than a year was similar to that binding of MDMA-naive controls. Moreover, Buchert et al. (2004) found a significant positive correlation between SERT binding and period of abstinence. Two years later, the same research group found a significant increase over the course of time of SERT binding of MDMA users and of SERT binding in the thalamus of ex-MDMA users, respectively (MDMA: *P* < 0.01; ex-MDMA: thalamus *P* = 0.006) (Buchert et al. 2006). Selvaraj et al. (2009) further supported the idea of SERT recovery; there was no difference in SERT binding between former ecstasy users and drug-naive controls after 1 year of abstinence. Moreover, Erritzoe et al. (2011) concluded that the duration of abstinence was positively related to SERT binding in pallidostriatum, amygdala and thalamus, but not in the neocortex. According to their data, recovery of the pallidostriatal SERT binding takes 200 days. In conclusion, there seems to be some evidence that there is a recovery of SERT binding. If there is indeed recovery of SERT binding over time, the relevant question is then whether this recovery represents functionally intact 5-HT neurons. The study of Reneman et al. (2001b) suggests that this may not be the case, because SERT binding in ex-MDMA users, who had stopped using MDMA for more than 1 year, was similar to control levels but demonstrated similar deficits on the RAVLT memory test as current MDMA users. Indeed, a couple of studies showed that even small doses of MDMA could lead to cognitive impairments, e.g. in verbal memory, and these impairments persist over time (Quednow et al. 2006; Schilt et al. 2007). A review of Parrott (2013) confirms that different cognitive functions can be affected by ecstasy use. There are deficits found not only in retrospective and prospective memory but also in higher cognition, complex visual processing, sleep architecture, sleep apnoea, pain, neurohormonal activity and psychiatric status. Therefore, recovery of SERT binding may reflect sprouting of 5-HT neurons or reduced endogenous neurotransmitter release after 5-HT toxicity has occurred, instead of recovery of the functional integrity of the 5-HT neurons.

However, another study of Halpern et al. (2011) found little evidence in ecstasy users for cognitive impairments. This study was designed to minimize methodological limitations and concluded that studies on cognitive function should be interpreted with caution. If it is true that use of ecstasy does not lead to persistent cognitive impairments, the recovery of SERT binding may simply reflect normalization of the adaptation (e.g. down-regulation), which may occur initially after MDMA use. In sum, several studies have shown that there is a recovery in SERT binding after MDMA use/administration, but it is not clear whether this is the result of recovery of the 5-HT neurons or other causes. Future fundamental studies on this topic are therefore recommended.

### Implications for practice

Selection criteria for the inclusion of subjects are very important for the quality of a given study. Research has shown that regular ecstasy users are polydrug users, so controls have to be matched on polydrug intake to rule out the effects of other drugs. As mentioned earlier, some studies showed that results were not significant anymore when polydrug-using controls were used instead of controls without a history of other drugs. The studies in this review used a great diversity of criteria to select subjects.

To generalize findings from animal studies to the human context, animal studies have to mimic the human context as accurately as possible. In many animal studies on the effects of MDMA, MDMA was administered passively. However, animal studies in which MDMA was self-administered may best reflect the human situation. The effects found on SERT binding were less pronounced in studies which used MDMA self-administration compared to studies which treated the animals passively with MDMA (ES ranged from −0.72 to 5.82 in SERT studies with self-administration and 0.69 to 20.03 in other studies), although the accumulated lifetime intake was higher in the studies with self-administering animals (97–141 mg/kg lifetime intake compared to 40–80 mg/kg in other studies). However, the number of animals used in these studies was relatively small; four MDMA self-administering monkeys and four controls were used in both studies.

## Concluding remarks

In the present review, we examined the effects of the use/administration of the drug ecstasy/MDMA on neurotransmitter systems in human and animal brains through imaging studies. The results of imaging studies reveal consistently that heavy use/administration of ecstasy/MDMA induces loss of SERT binding; however, these studies cannot conclude definitely whether this reduction in binding represents 5-HT neurotoxicity. The effects of MDMA/ecstasy on the 5-HT_2A_ system are not consistent, while in human, the DA system may not be significantly affected. Some studies showed that use of MDMA is correlated with deficits on several cognitive functions; however, opinions remain divided on this topic. Therefore, to come up with definite conclusions whether the use of ecstasy is neurotoxic in humans, large translational studies are still needed.

Current knowledge

- Heavy use/administration of MDMA decreases SERT binding; however, after a certain period of time (40–200 days), SERT binding recovers.
- In humans, MDMA does not seem to affect the DA system.
- Use of cannabis, cocaine and hallucinogens does not seem to influence the effects of MDMA on the SERT.

Remaining questions

- Is MDMA use able to induce detectable changes in 5-HT synthesis? To validate this, supporting studies in small laboratory animals may be performed with [^11^C]AMT PET.
- Does a decline in SERT binding reflect neurotoxicity? It may be relevant to perform more translational research.
- What is the cause and functional significance of SERT binding recovery; sprouting, regeneration, recovery of adaptation or endogenous neurotransmitter release? Future fundamental studies on this topic are recommended.
- What are the causes of an increase or decrease in receptor binding? Further research in animal brains could be done, e.g. using high-performance liquid chromatography to assess neurotransmitter concentrations and determination of *B*_max_ (number of binding sites) and *K*_d_ (affinity for the receptor).
- Does gender play an important role in susceptibility to possible toxic effects of MDMA use?
- What are the effects of young ecstasy use? Results suggest that there is an inverse relationship between age-at-first ecstasy use and [^123^ I]β-CIT binding ratios in the midbrain. However, only one study is performed on this topic; therefore, more research has to be done to draw valid conclusions.
